# Comparison of gemcitabine-based chemotherapies for advanced biliary tract cancers by renal function: an exploratory analysis of JCOG1113

**DOI:** 10.1038/s41598-021-92166-3

**Published:** 2021-06-18

**Authors:** Makoto Ueno, Chigusa Morizane, Takuji Okusaka, Junki Mizusawa, Tomoko Kataoka, Masafumi Ikeda, Masato Ozaka, Naohiro Okano, Kazuya Sugimori, Akiko Todaka, Satoshi Shimizu, Nobumasa Mizuno, Tomohisa Yamamoto, Keiji Sano, Kazutoshi Tobimatsu, Akio Katanuma, Atsushi Miyamoto, Hironori Yamaguchi, Tomohiro Nishina, Hirofumi Shirakawa, Yasushi Kojima, Takamasa Oono, Yasuyuki Kawamoto, Masayuki Furukawa, Tomohisa Iwai, Kentaro Sudo, Hiroyuki Miyakawa, Tatsuya Yamashita, Ichirou Yasuda, Hidenori Takahashi, Naoya Kato, Kazuhiko Shioji, Kyoko Shimizu, Toshio Nakagohri, Ken Kamata, Hiroshi Ishii, Junji Furuse, the members of the Hepatobiliary and Pancreatic Oncology Group of the Japan Clinical Oncology Group (JCOG-HBPOG).

**Affiliations:** 1 Department of Gastroenterology, Hepatobiliary and Pancreatic Medical Oncology Division, Kanagawa Cancer Center, 2-3-2 Nakao, Asahi ku, Yokohama, 241-0815 Japan; 2 Department of Hepatobiliary and Pancreatic Oncology, National Cancer Center Hospital, Chuo-ku, Tokyo Japan; 3 Japan Clinical Oncology Group/Operations Office, National Cancer Center Hospital, Chuo-ku, Tokyo Japan; 4 Department of Hepatobiliary and Pancreatic Oncology, National Cancer Center Hospital East, Kashiwa, Chiba Japan; 5 Hepato-Biliary-Pancreatic Medicine Department, Cancer Institute Hospital of Japanese Foundation for Cancer Research, Koto-ku, Tokyo Japan; 6 Department of Medical Oncology, Faculty of Medicine, Kyorin University, Mitaka, Tokyo Japan; 7 Gastroenterological Center, Yokohama City University Medical Center, Yokohama, Japan; 8 Division of Gastrointestinal Oncology, Shizuoka Cancer Center, Sunto-Gun, Shizuoka Japan; 9 Department of Gastroenterology, Saitama Cancer Center, Kita-Adachi-Gun, Saitama Japan; 10 Department of Gastroenterology, Aichi Cancer Center Hospital, Nagoya, Japan; 11 Department of Surgery, Kansai Medical University Hospital, Hirakata, Osaka Japan; 12 Department of Surgery, Teikyo University School of Medicine, Itabashi-ku, Tokyo Japan; 13 Division of Gastroenterology, Department of Internal Medicine, Kobe University Graduate School of Medicine, Kobe, Japan; 14 Center for Gastroenterology, Teine Keijinkai Hospital, Sapporo, Japan; 15 Department of Hepatobiliary and Pancreatic Surgery, Osaka National Hospital, Osaka, Japan; 16 Department of Surgery, Jichi Medical University, Shimono, Tochigi Japan; 17 Department of Gastrointestinal Medical Oncology, National Hospital Organization Shikoku Cancer Center, Matsuyama, Ehime Japan; 18 Department of Medical Oncology, Tochigi Cancer Center, Utsunomiya, Tochigi Japan; 19 Department of Gastroenterology, National Center for Global Health and Medicine, Shinjuku-ku, Tokyo Japan; 20 Department of Medicine and Bioregulatory Science, Graduate School of Medical Sciences, Kyushu University, Fukuoka, Japan; 21 Department of Gastroenterology and Hepatology, Hokkaido University Hospital, Sapporo, Japan; 22 Department of Hepato-Biliary-Pancreatology, National Hospital Organization Kyushu Cancer Center, Fukuoka, Japan; 23 Department of Gastroenterology, Kitasato University Hospital, Sagamihara, Kanagawa Japan; 24 Gastrointestinal Medical Oncology, Chiba Cancer Center, Chiba, Japan; 25 Department of Bilio-Pancreatology, Sapporo-Kosei General Hospital, Sapporo, Japan; 26 Department of Gastroenterology, Kanazawa University, Kanazawa, Ishikawa Japan; 27 Department of Gastroenterology and Hematology, Faculty of Medicine, University of Toyama, Toyama, Japan; 28 Department of Gastroenterological Surgery, Osaka International Cancer Institute, Osaka, Japan; 29 Department of Gastroenterology, Graduate School of Medicine, Chiba University, Chiba, Japan; 30 Department of Internal Medicine, Niigata Cancer Center Hospital, Niigata, Japan; 31 Department of Gastroenterology, Tokyo Women’s Medical University, Shinjuku-ku, Tokyo Japan; 32 Gastroenterological Surgery, Tokai University School of Medicine, Isehara, Kanagawa Japan; 33 Department of Gastroenterology and Hepatology, Faculty of Medicine, Kindai University, Osakasayama, Osaka Japan

**Keywords:** Biliary tract cancer, Chemotherapy

## Abstract

JCOG1113 is a randomized phase III trial in patients with advanced biliary tract cancers (BTCs) (UMIN000001685), and gemcitabine plus S-1 (GS) was not inferior to gemcitabine plus cisplatin (GC). However, poor renal function often results in high toxicity of S-1. Therefore, we examined whether GS can be recommended for patients with low creatinine clearance (CCr). Renal function was classified by CCr as calculated by the Cockcroft-Gault formula: high CCr (CCr ≥ 80 ml/min) and low CCr (80 > CCr ≥ 50 ml/min). Of 354 patients, 87 patients on GC and 91 on GS were included in the low CCr group, while there were 88 patients on GC and 88 patients on GS in the high CCr group. The HR of overall survival for GS compared with GC was 0.687 (95% CI 0.504–0.937) in the low CCr group. Although the total number of incidences of all Grade 3–4 non-haematological adverse reactions was higher (36.0% vs. 11.8%, *p* = 0.0002), the number of patients who discontinued treatment was not different (14.1% vs. 16.9%, *p* = 0.679) for GS compared with GC in the low CCr group. This study suggests that GS should be selected for the treatment of advanced BTC patients with reduced renal function.

## Introduction

Biliary tract cancers (BTCs) include those in the intrahepatic bile duct (IHBD), extrahepatic bile duct (EHBD), gallbladder (GB), and ampulla of Vater (AV). Although the incidence of BTC is low, it varies according to geographic region. In Japan, BTCs other than IHBD are the 6th leading cause of cancer death, with approximately 18,000 patients^1^, and BTCs are generally diagnosed at an advanced stage^2^. Gemcitabine plus cisplatin (GC) has been the standard chemotherapy treatment for advanced/recurrent BTCs since 2010^3^. Recently, ABC-06 showed the superiority of FOLFOX compared with active symptom control as a second-line treatment^4^. As targeted agents, inhibitors of fibroblast growth factor receptor (FGFR) aberrations, such as pemigatinib and futibatinib, showed promising activities in the IHBD^5,6^. Moreover, immune checkpoint inhibitors are being actively developed^7–10^. However, the recommended treatment options for unresectable or metastatic disease have been limited, and the prognosis of these patients is poor, with a median overall survival (OS) of approximately 1 year. S-1, an oral fluoropyrimidine combination drug consisting of tegafur, gimeracil, and oteracil, has achieved promising outcomes for several cancers^11,12^.

The Japan Clinical Oncology Group (JCOG) Hepatobiliary and Pancreatic Oncology Study Group conducted a randomized phase III trial to confirm that gemcitabine plus S-1 (GS) is not inferior to GC for unresectable or recurrent BTCs (JCOG1113). From May 2013 to March 2016, 350 patients were registered for JCOG1113. In the final analysis, GS was demonstrated not to be inferior to GC for OS (median OS, 13.4 months with GC and 15.1 months with GS; hazard ratio [HR] 0.945; 90% confidence interval [CI] 0.78–1.15; *P* = 0.046 for non-inferiority), with good tolerability, and was considered to be a new convenient treatment option without hydration for advanced BTCs.

Renal function needs to be considered when using cisplatin, because of its renal toxicity^13^. Furthermore, gimeracil, one of the components of S-1, inhibits the metabolism of fluorouracil and is excreted in urine^14^. The serum concentration of gimeracil increases in patients with renal dysfunction, which theoretically results in a higher serum concentration of fluorouracil. Based on this mechanism, previous studies examined the influence of renal function on the toxicity of S-1 monotherapy (80 mg/day for a body surface area [BSA] on days 1–28 followed by a 14-day rest); patients with low creatinine clearance (CCr) showed a high incidence of severe adverse events (AEs) that resulted in treatment discontinuation^15–17^. Although there are no reports that have evaluated the influence of renal function on survival outcomes for patients with advanced BTCs, high incidence of AEs may result in a poor prognosis due to a low dose intensity. However, AEs and survival outcomes for patients treated with GS have not been reported. Therefore, we examined whether GS can be recommended for patients with low CCr in JCOG1113 as an exploratory analysis.

## Methods

### Summary of JCOG1113

JCOG1113 is a multicentre open-label randomized phase III study that was conducted to confirm that GS is not inferior to GC for OS in patients with advanced/recurrent BTCs. The key eligibility criteria for JCOG1113 were as follows: histologically proven BTCs (adenocarcinoma or adenosquamous carcinoma of the IHBD, EHBD, GB, or AV), unresectable or recurrent disease, age 20–79 years, Eastern Cooperative Oncology Group performance status score of 0 or 1, adequate self-supported nutritional intake, no previous treatment for BTCs except surgery or biliary drainage, no previous chemotherapy or radiotherapy, adequate function of the major organs, serum creatinine ≤ 1.2 mg/dl, and CCr ≥ 50 ml/min.

Patients assigned to GC received gemcitabine (1000 mg/m^2^) and cisplatin (25 mg/m^2^) infusion on days 1 and 8; this regimen was repeated every 3 weeks. Cisplatin was administered up to 16 times (400 mg/m^2^) unless patients met the termination criteria. After cisplatin termination, patients received gemcitabine (1000 mg/m^2^) infusion on days 1, 8, and 15, and this was repeated every 4 weeks. Patients assigned to GS received gemcitabine (1000 mg/m^2^) infusion on days 1 and 8. S-1 was administered orally twice daily (60 mg/day for a BSA < 1.25 m^2^, 80 mg/day for a BSA between 1.25 and 1.50 m^2^, and 100 mg/day for a BSA 1.50 m^2^) on days 1–14. This regimen was repeated every 3 weeks.

### Patients

The secondary use of data from JCOG1113 was included in the written informed consent provided by all patients, and approval was obtained from the Institutional Review Board of 32 participated institution (Kanagawa Cancer Center, National Cancer Center Hospital, National Cancer Center Hospital East, Cancer Institute Hospital of Japanese Foundation for Cancer Research, Kyorin University Faculty of Medicine, Yokohama City University Medical Center, Shizuoka Cancer Center, Saitama Cancer Center, Aichi Cancer Center Hospital, Kansai Medical University Hospital, Teikyo University School of Medicine, Kobe University Graduate School of Medicine, Teine Keijinkai Hospital, Osaka National Hospital, Jichi Medical University, National Hospital Organization Shikoku Cancer Center, Tochigi Cancer Center, National Center for Global Health and Medicine, Kyushu University, Hokkaido University Hospital, National Hospital Organization Kyushu Cancer Center, Kitasato University Hospital, Chiba Cancer Center, Sapporo-Kosei General Hospital, Kanazawa University, University of Toyama, Osaka International Cancer Institute, Chiba University, Niigata Cancer Center Hospital, Tokyo Women's Medical University, Tokai University School of Medicine, and Kindai University Faculty of Medicine). All eligible patients registered in JCOG1113 (n = 175/179 in the GC/GS arm) were included in this analysis. These patients were divided according to renal function. The study was conducted in accordance with the ethical standards established in the 1964 Declaration of Helsinki and its later amendments. All patients provided written informed consent.

### Statistical analysis

AEs related to the treatment were defined as adverse reactions (ARs). ARs and AEs were reported according to Common Terminology Criteria for Adverse Events (CTCAE) version 4.0 in JCOG1113. In the present study, we defined the number of ≥ Grade 3 ARs as a mark of safety. OS was defined as the duration from the date of registration in the trial to the date of any cause of death or final follow-up. Progression-free survival (PFS) was defined as the duration from the date of registration in the trial to the date of documented disease progression or any cause of death. Response rate (RR) was defined as the proportion of CR or PR patients according to Response Evaluation Criteria in Solid Tumors version 1.1. Percentage of planned dose was defined as the ratio of the delivered dose to the planned dose.

The endpoints of the present study were OS, PFS, ≥ Grade 3 AR, and the percentage of planned dose. To examine the effects of renal function on these endpoints, we classified patients into high CCr (≥ 80 ml/min) and low CCr (< 80 ml/min) groups relative to the median value. CCr was calculated using the Cockcroft-Gault formula^18^ or was obtained from the actual data at the time of registration. The incidences of ARs and response rate were evaluated in each subgroup, and Clopper-Pearson 95% confidence intervals (95% CIs) were calculated. OS and PFS were estimated using the Kaplan–Meier method, and 95% CIs of the median OS and PFS were evaluated using the methods of Brookmeyer and Crowley. To assess the prognostic difference of each factor for OS and PFS, the HRs and 95% CIs were estimated using Cox’s regression model, and log-rank p-values were calculated. The percentages of planned doses for gemcitabine, cisplatin, and S-1 were expressed using the median values. These were compared with the Wilcoxon rank-sum test. ARs, total numbers of incidences of all Grade 3–4 non-haematological ARs, the numbers of patients who discontinued treatment and RRs were compared using Fisher’s exact test. All statistical analyses were performed with SAS version 9.4 (SAS Institute, Cary, NC) at the JCOG Data Center.

## Results

There were 87 and 88 patients in the low and high CCr groups, respectively, in the GC arm and 91 and 88 patients in the low and high CCr groups, respectively, in the GS arm. Patient characteristics in the high and low CCr groups are shown in Table 1. Median age and the percentage of female patients were higher in the low CCr group.

**Table 1:** Patient characteristics in the high and low CCr groups (n = 354).

	Low CCr (< 80 ml/min)			High CCr (≥ 80 ml/min)		
	GC n = 87	GS n = 91	Total n = 178	GC n = 88	GS n = 88	Total n = 176
Age (years)						
Median	70	73		65	63	
Interquartile Range (IQR)	66–73	67–76		58–63	57–68	
Sex, n (%)						
Male	42 (48.3)	44 (48.4)		57 (64.8)	53 (60.2)	
Female	45 (51.7)	47 (51.6)		31 (35.2)	35 (39.8)	
ECOG PS, n (%)						
0	63 (72.4)	61 (67.0)		67 (76.1)	63 (71.6)	
1	24 (27.6)	30 (33.0)		21 (23.9)	25 (28.4)	
Primary site, n (%)						
Gallbladder	32 (36.8)	35 (38.5)		36 (40.9)	34 (38.6)	
IHBD	28 (32.2)	21 (23.1)		22 (25.0)	23 (26.1)	
EHBD	23 (26.4)	31 (34.1)		26 (29.5)	28 (31.8)	
Perihilar	12 (13.8)	16 (17.6)		17 (19.3)	16 (18.2)	
Distal	11 (12.6)	15 (16.5)		9 (10.2)	12 (13.6)	
Ampulla of Vater	4 (4.6)	3 (3.3)		3 (3.4)	3 (3.4)	
Other (ineligible)		1 (1.1)		1 (1.1)		
Biliary drainage, n (%)						
Present	35 (40.2)	41 (45.1)		37 (42.0)	41 (46.6)	
Stage, n (%)						
M0	16 (18.4)	15 (16.5)		15 (17.0)	17 (19.3)	
M1	50 (57.5)	57 (62.6)		56 (63.6)	50 (56.8)	
Recurrence	21 (24.1)	18 (19.8)		16 (18.2)	21 (23.9)	
White blood cell						
Median	5900	6110		5800	5355	
IQR	4900–7400	4930–8100		4860–7550	4490–7350	
Albumin						
Median	3.7	3.7		3.9	3.90	
IQR	3.4–4.1	3.3–4.0		3.6–4.1	3.40–4.15	

*CCr* creatinine clearance, *ECOG PS* Eastern Cooperative Oncology Group performance status, *EHBD* extrahepatic bile duct, *GC* gemcitabine plus cisplatin, *GS* gemcitabine plus S-1, *IHBD* intrahepatic bile duct, *IQR* interquartile range.

### Safety

The incidences of clinically important Grade ≥ 3 ARs in the high and low CCr groups are shown in Table 2. The incidences of Grade ≥ 3 haematological ARs were higher in the low CCr group in each regimen, respectively. Looking at each CCr group, the incidence of Grade ≥ 3 non-haematological ARs was higher in the GS arm (36.0%; 95% CI 26.1–46.8%) than that in the GC arm (11.8%; 95% CI 5.8–20.6%) in the low CCr group (*p* = 0.0002). On the other hand, the incidences of Grade ≥ 3 haematological ARs, including white blood cell count decreased (35.3%/23.6%), anaemia (29.4%/7.9%), and platelet count decreased (18.8%/10.1%), were higher in the GC arm than those in the GS arm in the low CCr group. The number of patients who discontinued treatment was not different (14.1%/16.9%, *p* = 0.679) in the GS arm compared with the GC arm in the low CCr group. No remarkable significant differences were observed in the percentages of planned doses for gemcitabine, cisplatin, and S-1 (median), as shown in Table 3.

**Table 2:** Incidence of clinically important ≥ Grade 3 adverse reactions in the high and low CCr groups.

CTCAE ver. 4.0, n (%)	Low CCr		High CCr	
	GC (n = 85)	GS (n = 89)	GC (n = 86)	GS (n = 88)
Haematological ARs				
White blood cell decreased	30 (35.3)	21 (23.6)	23 (26.7)	23 (26.1)
Anaemia	25 (29.4)	7 (7.9)	12 (14.0)	3 (3.4)
Platelet count decreased	16 (18.8)	9 (10.1)	12 (14.0)	4 (4.6)
Neutrophil count decreased	52 (61.2)	50 (56.2)	52 (60.5)	56 (63.6)
Non-haematological ARs				
Diarrhoea	0	0	0	1 (1.1)
Mucositis oral	0	3 (3.4)	0	0
Palmar-plantar erythrodysesthaesia syndrome	0	1 (1.1)	0	1 (1.1)
Biliary tract infection	4 (4.7)	9 (10.1)	6 (7.0)	4 (4.5)
Fatigue	4 (4.7)	5 (5.6)	0	3 (3.4)
Fever	0	0	1(1.2)	0
Nausea	2 (2.4)	2 (2.2)	0	1 (1.1)
Vomiting	2 (2.4)	1 (1.1)	0	0
Anorexia	5 (5.9)	4 (4.5)	2 (2.3)	4 (4.5)
Febrile neutropenia	1 (1.2)	3 (3.4)	3 (3.5)	1 (1.1)
Total number of non-haematological ARs	10 (11.8)	32 (36.0)	11 (12.8)	16 (18.2)

*AR* adverse reaction, *CCr* creatinine clearance, *CTCAE* Common Terminology Criteria for Adverse Events, *GC* gemcitabine plus cisplatin, *GS* gemcitabine plus S-1.

**Table 3:** Percentage of planned dose (median, IQR; %).

	Low CCr		High CCr	
	GC	GS	GC	GS
Gemcitabine	75.0 (60.4–92.9)	85.7 (65.0–94.4)	75.0 (62.5–89.9)	75.0 (50.0–92.7)
Cisplatin	75.0 (63.6–92.9)		75.7 (63.6–91.7)	
S-1		82.9 (65.0–92.9)		74.4 (50.0–93.8)

*CCr* creatinine clearance, *GC* gemcitabine plus cisplatin, *GS* gemcitabine plus S-1, *IQR* interquartile range.

### Efficacy

The response rates for GC and GS were 32.1% and 32.9%, respectively, in the low CCr group. In the high CCr group, they were 32.9% and 26.5%, respectively.

**Figure 1:**
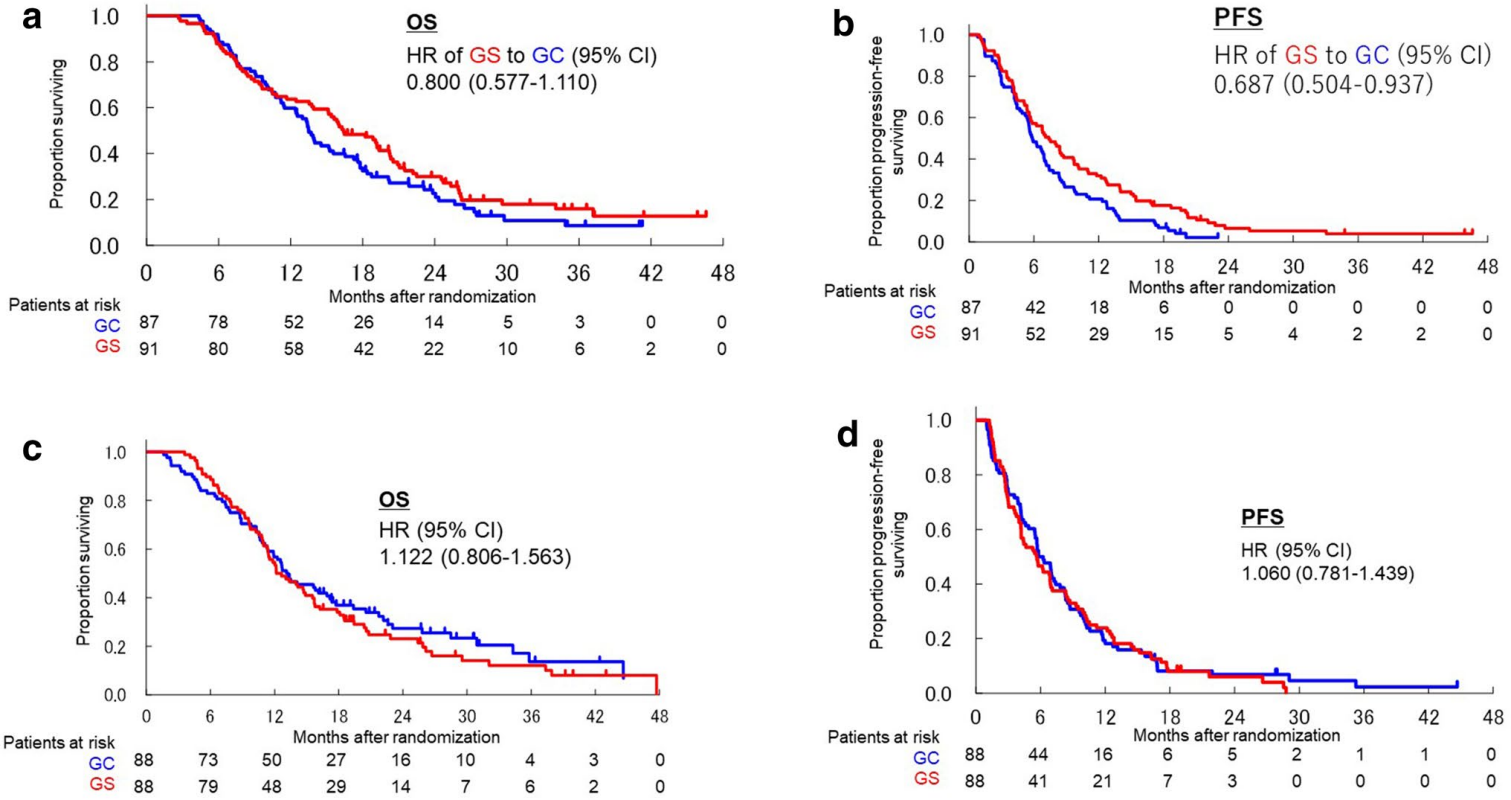
Comparison of overall survival and progression-free survival in each CCr group according to GC and GS arm. (**a**) Kaplan–Meier curves of overall survival in the low CCr group. (**b**) Kaplan–Meier curves of progression-free survival in the low CCr group. (**c**) Kaplan–Meier curves of overall survival in the high CCr group. (**d**) Kaplan–Meier curves of progression-free survival in the high CCr group. CCr, creatinine clearance; CI, confidence interval; GC, gemcitabine plus cisplatin; GS, gemcitabine plus S-1; HR, hazard ratio; OS, overall survival; PFS, progression-free survival.

Kaplan–Meier curves for OS and PFS in each CCr group are shown in Fig. 1a–d. The HRs of OS and PFS for GS compared with GC were 0.800 (95% CI 0.577–1.110) and 0.687 (95% CI 0.504–0.937), respectively, in the low CCr group. They were 1.122 (95% CI 0.806–1.563) and 1.060 (95% CI 0.781–1.439), respectively, in the high CCr group.

**Figure 2:**
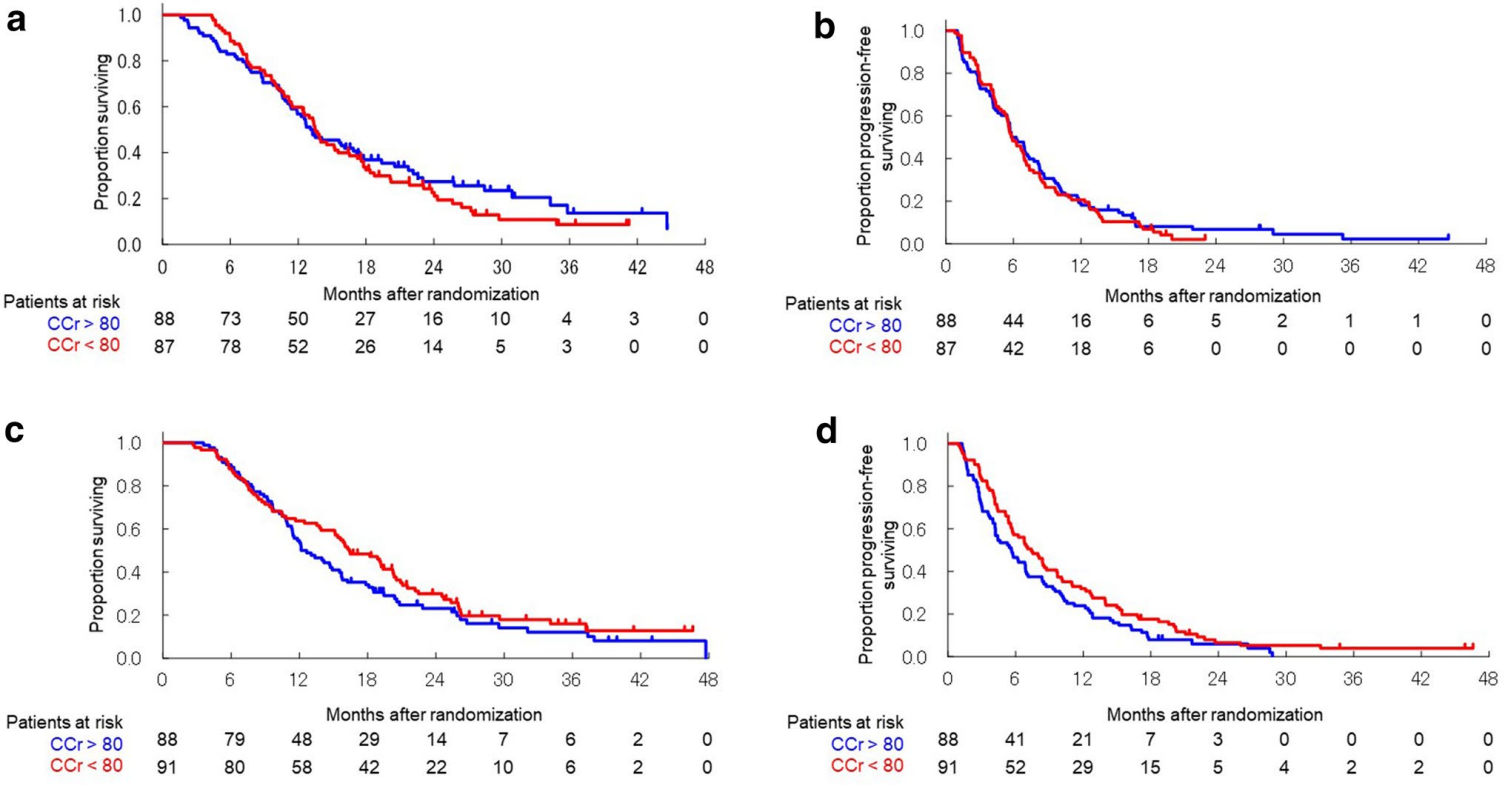
Comparison of overall survival and progression-free survival in the GC and GS arms according to the low and high CCr groups. (**a**) Kaplan–Meier curves of overall survival for GC. (**b**) Kaplan–Meier curves of progression-free survival for GC. (**c**) Kaplan–Meier curves of overall survival for GS. (**d**) Kaplan–Meier curves of progression-free survival for GS. CCr, creatinine clearance; CI, confidence interval; GC, gemcitabine plus cisplatin; GS, gemcitabine plus S-1.

Kaplan–Meier curves for OS and PFS in each regimen are shown in Fig. 2a–d. The HRs of OS and PFS for low CCr compared with high CCr were 1.112 (95% CI 0.804–1.556) and 1.081 (95% CI 0.796–1.468), respectively, for GC. The HRs of OS and PFS for low CCr compared with high CCr were 0.806 (95% CI 0.582–1.117) and 0.749 (95% CI 0.553–1.013), respectively, for GS.

A multivariable analysis of OS and PFS revealed that the HRs for GS compared with GC in the low CCr group were 0.775 (95% CI 0.577–1.078; *P* = 0.13) and 0.658 (95% CI 0.479–0.906; *P* = 0.01), respectively, as shown in Table 4.

**Table 4:** Multivariable analysis of clinicopathological factors for PFS and OS in the low CCr group.

Factor	PFS		OS	
	Hazard ratio (95% CI)	*p*-value^a^	Hazard ratio (95% CI)	*p*-value^a^
Chemotherapy				
GC	1		1	
GS	0.658 (0.479–0.906)	0.01	0.775 (0.557–1.078)	0.13
Age (years)				
< 67	1		1	
≥ 67	0.834 (0.573–1.215)	0.35	1.058 (0.710–1.576)	0.78
Sex				
Male	1		1	
Female	0.986 (0.709–1.372)	0.93	0.971 (0.689–1.368)	0.87
ECOG PS				
0	1		1	
1	1.395 (0.999–1.948)	0.05	1.299 (0.907–1.858)	0.15
Primary site				
Gallbladder	1		1	
Others	0.775 (0.558–1.077)	0.129	0.828 (0.587–1.167)	0.28
History of curative resection				
None	1		1	
Present	1.142 (0.782–1.668)	0.492	0.839 (0.557–1.265)	0.40

*CCr* creatinine clearance, *CI* confidence interval, *ECOG PS* Eastern Cooperative Oncology Group performance status, *GC* gemcitabine plus cisplatin, *GS* gemcitabine plus S-1, *OS* overall survival, *PFS* progression-free survival.

^a^Two-sided.

## Discussion

This is the first study to compare the influence of CCr on the efficacy and safety of GC and GS in BTCs. In the future, the development of targeted agents and immune checkpoint inhibitors will progress along with that of the combination of cytotoxic agents. Thus, it is still important to understand the characteristics of the combination of cytotoxic agents. The low CCr group (< 80 ml/min) showed better PFS only in the GS arm. Moreover, response rates in the GS arm were higher in the low CCr group, and the HRs of OS and PFS for the low CCr group were better in the GS arm. Regarding safety, although the incidence of non-haematological Grade ≥ 3 ARs was higher in the GS arm than that in the GC arm, the toxicity of GS was not severe in the low CCr group. Therefore, GS may be recommended for the low CCr group.

These results may be attributed to a higher serum concentration of fluorouracil caused by gimeracil due to renal dysfunction in the low CCr group compared with the serum concentration in the high CCr group. As for efficacy, a high S-1 dose intensity is reported to be related to better results in gastric cancer^19^. GS in the low CCr group would have the same high S-1 dose intensity. With regard to safety, previous studies reported that renal dysfunction was associated with increases in toxicities. In the present study, increases were observed in total ≥ Grade 3 non-haematological ARs. However, the percentage of planned dose for S-1 was similar in both the low and high CCr groups, which was interpreted as good tolerability of the GS regimen despite reduced renal function. This may be attributed to the administration schedule and dose of S-1. S-1 is generally administered for 4 weeks followed by a 2-week rest. In the present GS therapy, the dose of S-1 administered was lowered by one level for 2 weeks followed by a 1-week rest. Toxicity is usually greater with administration for 4 weeks followed by a 2-week rest compared with two cycles of 2 weeks of administration followed by a 1-week rest^20^. Thus, in patients with renal dysfunction, S-1 may sometimes enhance antitumor effects and at other times show an increase in toxicity.

The percentage of planned dose for S-1 is an important factor in BTC patients treated with GS. In the present study, the percentage of planned dose for S-1 was not lower in the low CCr group than in the high CCr group despite the predicted higher toxicities. Regarding maintenance of the percentage of planned dose for S-1 in the low CCr group, the S-1 dosage could be determined based on renal function as well as BSA, as reported previously^21^. GS with a one level higher dose of S-1 (80 mg/m^2^ BSA per day on days 1–14) in the high CCr group may be a possibility. Previously, a phase II study in pancreatic cancer patients examined GS with a daily S-1 dose of 80 mg/m^2^ BSA (on days 1–14), and it showed a high response rate and modest toxicities^22^.

On the other hand, low CCr did not affect the efficacy and safety of GC. The renal toxicity of cisplatin is well known; however, it is accumulated toxicity. This study limited the total volume of cisplatin to ≤ 400 mg/m^2^/BSA, and each volume of cisplatin was 25 mg/m^2^/BSA. No large increases in creatine were observed for GC, at least with a total volume of cisplatin of < 400 mg.

This exploratory study had some limitations. Since this was an exploratory analysis, it did not have sufficient statistical precision. Although a prospective phase III study in patients with low CCr is necessary, this type of study is difficult to perform in patients with advanced/recurrent BTCs. In addition, this analysis was limited to patients with CCr ≥ 50 ml/min. The present analysis should not be used for patients with CCr < 50 ml/min. Moreover, the CCr value in most patients is calculated using the Cockcroft-Gault formula. This formula has a tendency to calculate a lower value for CCr than the actual value in elderly and female patients. Furthermore, this study included four sites as BTCs (gallbladder, IHBD, EHBD, ampulla of Vater). The number of patients for each site was limited, and the results should be interpreted carefully. In addition, the study was limited to an Asian population, and it is not possible to extrapolate the results directly to populations in western countries.

In conclusion, GS seemed to be superior to GC in terms of OS and PFS in the low CCr group. As for the percentage of planned dose for S-1, there was no remarkable difference between the two CCr groups despite the higher proportion of non-haematological ARs (≥ Grade 3) in the low CCr group. This study suggests that GS should be selected for the treatment of advanced BTC patients with reduced renal function.

## Data Availability

The authors confirm that the data supporting the findings of this study are available within the article.
